# Reflect to interact – fostering medical students’ communication through reflection-focused e-learning

**DOI:** 10.1186/s12909-024-05368-4

**Published:** 2024-05-15

**Authors:** Laura Janssen, Kristina Schick, Tiziana Neurohr, Sylvia Irene Donata Pittroff, Sabine Reiser, Johannes Bauer, Pascal O. Berberat, Martin Gartmeier

**Affiliations:** 1 Department Clinical Medicine - TUM School of Medicine and Health, Technical University of Munich, TUM Medical Education Center, Munich, Germany; 2 Educational Research and Methodology, University of Erfurt, Erfurt, Germany

**Keywords:** Communication, e-learning, Medical education, Online learning, Reflective practice, Reflective writing

## Abstract

**Background:**

Previous research indicates that reflection can foster medical communication competence. However, best practices for embedding reflection in online medical education are missing. This study examined how reflection processes can be promoted and embedded in an e-learning course on physician–patient communication to foster learning.

**Methods:**

We investigated three differently designed e-learning conditions featuring different proportions of reflection triggers and compared their effects on students’ reflections. We had 114 medical students in their first clinical year complete one of the variants: video modelling (VM, *n* = 39), video reflection (VR, *n* = 39), or a variant merging both approaches (VMR, *n* = 36). Each student wrote a total of nine reflections based on the same guiding questions at three time points embedded in the course. The students’ levels of reflection were measured using an adapted version of the REFLECT rubric (scale 0–18).

**Results:**

Students of all course variants achieved good levels of reflection beyond the descriptive level at all three time points, with no significant differences between the variants. The mean reflection scores at the end of the course were *M* = 14.22 for VM (*SD* = 2.23), *M* = 13.56 for VR (*SD* = 2.48), and *M* = 13.24 for VMR (*SD* = 2.21). Students who completed VM showed significantly improved levels of reflection over the course, whereas we found no statistically significant development for those in VR or VMR. The reflection scores correlated significantly positively with each other, as did the text lengths of the written reflections. Reflection scores also correlated significantly positively with text length.

**Conclusions:**

Our study offers a teaching strategy that can foster good levels of reflection, as demonstrated in the three e-learning variants. The developed reflection prompts can be easily embedded in various e-learning environments and enable reflections of good quality, even in settings with limited available teaching time.

**Supplementary Information:**

The online version contains supplementary material available at 10.1186/s12909-024-05368-4.

## Background

Interaction with patients is fundamental to medical practice [1–4] and providing quality care [5, 6]. Physician–patient interactions have an impact on patients, physicians, and the relationship between them [7]. It affects patients’ health [8, 9] as well as physicians’ health (stress level and burnout risk) [4, 10]. Problems during physician–patient interactions also cause a major part of patients’ complaints [2]. Hence, medical students must develop clinical communication competence [6], which must go beyond familiarity with basic theoretical concepts or being able to reiterate phrases commonly used in interactions. Prospective physicians have to adapt their communication [11] in a context- and situation-specific way to the individual patient [6]. They must also develop sensitivity to their verbal and non-verbal expressions, integrate the patient into the conversation, and be able to cope with emotions [12].

As medical education shifts from a transmissive (teacher-centred) to a reflective learning model [13] inspired by constructivist learning theory [14], reflection is increasingly incorporated into curricula [15]. To develop individual communication strategies, students should reflect on (observed or their own) experiences with patients [16]. Reflection as a “metacognitive process creates a deeper understanding of the self and the situation to inform future actions” [17]. By critically analysing a situation and their cognitive and behavioural responses to the situation [16], students can develop self-awareness and awareness of the other and the situation itself [12]. They might identify new perspectives and lessons learned that they can apply in future interactions [18, 19]. Implementing reflection in clinical communication training may increase students’ communicative competency [5], as reflection might correlate with communication skills [20]. Furthermore, reflection in general can improve skills in health care [21], particularly the quality of patient care [22], and may enhance empathy among practicing physicians [23, 24]. Therefore, good medical communication training should encourage students to reflect [6].

Extending digitalisation is another shift in medical education, creating the momentum to design new teaching approaches [4] and offering new learning opportunities for medical students [13]. Studies have indicated the effectiveness of online learning in fostering the communication skills of health care providers. Learning with online modules was shown to increase the ability to break bad news [25] and enhance physiotherapy students’ ability to handle distressing communication within a single e-learning training [26], and it was more effective than role-play [27]. Further, e-learning offers the possibility of individualised learning pathways, for example, learning pathways adapted to the needs of advanced learners by providing additional content [28]. However, as a promising teaching approach within medical education [29], e-learning bears unique characteristics, opportunities, and challenges. In self-study, students can flexibly adjust the time they spend with the teaching materials. However, students often cannot ask direct questions, and individual feedback is usually delayed. Additionally, lecturers do not get an impression of their students during delivery. Therefore, it is essential to ensure that while learning autonomously, students deal with course content in a way that leads to effective learning and improved competence. Collecting evidence on promising learning environment designs and teaching approaches of online courses is essential to inform clinical teachers about how to foster the effective learning and competence development of their students.

In this respect, we consider initiated reflections embedded in e-learning formats to be particularly promising. During reflection, students actively deal with the course content. As medical education is moving towards more reflection-orientated learning, e-learning should also include methods that promote critical thinking skills and reflective practice [13]. However, there is limited research on encouraging learning environments or pedagogical strategies to enhance medical students’ reflection [30], and despite the known benefits of reflection in various health care professions, it is still not a thriving practice in medical education [31]. Various authors have described how to measure reflection, but only a few have addressed how to embed reflection into online teaching [32]. Although reflection prompts have been described in recent literature, evidence of the suspected effects remains limited [33]. Best practices are rarely known, and acceptable approaches to teaching reflection or consistent guiding principles do not yet exist [34–37].

The aim of this study was to examine how reflection processes can be embedded and promoted in an e-learning course on medical communication competence. Therefore, our study explores how differently designed e-learning conditions foster reflection, as well as how reflection can be embedded in courses in which little time is available for reflection. Hence, we also aimed to gain initial insights into whether short written reflections can promote reflective capacity. Understanding how reflection could be facilitated to foster medical students’ communication, considering the specific characteristics of online learning, might offer new teaching strategies and improve student learning.

We designed three e-learning variants of a course on medical communication competence with different proportions of reflection triggers and analysed students’ written reflections, as they have been proven to encourage deep reflective thinking [38, 39]. Based on an exploratory approach without formulating hypotheses, we aimed to answer the following research questions:

(i) Which e-learning course variant is most effective in reflecting on the course content: a teaching approach that focuses on independently deducing the course content (VR), an explanatory teaching approach based on video modelling using good practice examples and exemplifications (VM), or a variant merging both approaches (VMR)?
(ii) Which levels of reflection are reached in the course variants?
(iii) Does the quality of reflection correlate with students’ written text length?

## Methods

We redesigned a face-to-face introductory course on medical communication for medical students in their first clinical year into three e-learning variants, using the course management system Moodle. All variants comprised the same theoretical content, including videos of simulated physician–patient interactions. The variants differed in their proportion of learning elements fostering reflections (reflection triggers) on course content and own learning process. The number of reflection triggers increased in the variants (0, 1, and 2 triggers). In the variant with two triggers, two different triggers were used to promote reflection (Table 1). All three course modules followed the same structure.

**Table 1:** Outline of the three course variants for Module 2: Structuring consultations

Video modelling (VM)	a mixture of VM and VR (VMR)	Video reflection (VR)
Whiteboard presentation on theory	Students write a non-guided reflection about the module theme*	Students write a non-guided reflection about the module theme*
Good practice videos	Whiteboard presentation on theory	Whiteboard presentation on theory
Quiz	Quiz	Quiz
Video example Entire physician–patient conversation accompanied by expert feedback and explanatory statements on video examples	Video example Entire physician–patient conversation accompanied by expert feedback and explanatory statements on video examples	**Interactive exercise** Entire physician–patient conversation selected in eight parts; students have to identify the optimal structure.*
Take-home message	Take-home message	Take-home message
Reflection prompts based on three guiding questions	Reflection prompts based on three guiding questions	Reflection prompts based on three guiding questions

*Reflection trigger

We designed the variant without reflection triggers based on *video modelling* (VM) [27, 40] using an illustrative example (rule-example approach) [41], which has been proven in previous research to promote factual knowledge [42, 43]. This variant focused on explaining good and bad practice videos. For example, students watched an entire physician–patient consultation in the correct order complemented by explanations. The level of complexity to which learners were exposed was limited, and close guidance was offered [41, 43].

In contrast to this teaching approach, we designed another variant with two reflection triggers, which we called *video reflection* (VR) [41]. This variant included one interactive video exercise in each module (m), as videos—particularly interactive video exercises—have been described as a promising approach to stimulating written reflection in previous research [18, 44]. VM focuses on interactive engagement with the same videos as in the variant VM but without offering explanations. For example, the above-mentioned physician–patient consultation was segmented, and students had to select the optimal structure by themselves. Interaction is described as a critical factor in terms of how the material is presented to capture learners’ attention and effectively facilitate student learning transfer [45]. Thus, interaction can encourage engagement with the learning material and has become one of the most important strategies in e-learning to teach critical thinking [46], which is associated with reflective capacity [47]. In addition to the interactive video exercise, we used an open-ended question at the beginning of each module as a second reflection trigger, asking the students how the exercise was associated with the course topic of this module. Students had to compose a short text before they could proceed in the course module.

The third variant (VMR) combined elements from both approaches. It contained one reflection trigger—the open-ended question about the association of the exercise with the module topic.

Each course variant contained three 45-minute modules on *the basics of communication* (Module 1), *structuring consultations* (Module 2), *and empathy and emotions* (Module 3). The e-learning course was moderated by two physicians (female and male) with specialisations in gynaecology and psychosomatic medicine, who conveyed the same theoretical content to all students, while different teaching approaches were used in the e-learning variants. Learning was asynchronous in all variants. The physician–patient interactions shown in the videos focused on the topics “basics of communication” and “beginning a physician–patient interaction” (Module 1); “conducting and structuring an entire physician–patient conversation with a new patient (in the medical field of internal medicine)” (Module 2); and “reacting appropriately to the emotions of stressed patients” (Module 3). At the end of each module, the same written reflections were prompted in every variant (Table 1). By employing guiding questions, the students reflected on the course content and their learning processes. Based on their reflections, we examined how actively students dealt with the learning material and how deeply they reflected, as well as which course variant increased the quality of the reflections.

### Reflection prompts

The reflection prompts chosen to initiate the written reflections were based on Koole et al. [18] and focused on the three core elements of reflection: *awareness*, *understanding*, and *future actions/transfer* [48–50]. Koole et al. used six guiding questions to assess the quality of medical students’ reflections. These questions made the three core elements of reflection visible and distinctly measurable, although they are usually merged within a reflection process. In contrast to Koole et al., we did not pose questions explicitly asking about emotions, as we aimed to explore whether students reflected on emotions on their own initiative. We adapted the questions in the following way:

1. Reflection prompt: **Awareness**
  Describe some aspects that you noticed during the physician?patient interactions shown in the videos
2. Reflection prompt: **Understanding**
  What did you learn? How? Why is that useful?
3. Reflection prompt: **Impact on your future physician–patient interactions**
  Which learnings might be helpful for your future work? What do you plan for your physician?patient interactions?

We embedded these questions in all variants at the end of a course module (three time points). Therefore, students answered each question three times, as recent literature indicates that a single sample is insufficient for an accurate assessment of reflectivity [51]. The students had to answer the questions to proceed in the course. A minimum of 500 characters per prompt was specified to promote the reflection process. There was no time limit. Furthermore, the written reflections were not graded to minimise extrinsic motivations [52].

### Participants

Data were collected in the winter semester 2020/21 at the Technical University of Munich. The study participants were medical students in their first clinical year enrolled in a curricular, obligatory course on medical communication. Thereby, effects due to possibly above-average motivated, voluntarily participating students were reduced [53]. A total of 121 students were randomly assigned to one of the three course variants, with 114 completing all modules and reflection prompts (VM: *n* = 39, VR: *n* = 39, VMR: *n* = 36, 80 female, 33 male, 1 other). The average age was 22.02 years (*SD* = 2.48). How students processed the e-learning environment was flexible regarding location and time. The students had not previously received lessons about reflective thinking.

### Data analysis

We applied the REFLECT (Reflection Evaluation for Learners’ Enhanced Competencies) rubric, as it is an established tool for assessing reflective capacity [16, 37] widely used in medical education [16, 19, 54]. The rubric developed by Wald et al. measures the depth of reflection. It comprises five criteria that assess five core processes of reflection [55]: the *spectrum of written exploration*, the *writer’s presence* in the written work, the quality of *description of conflict or disorienting dilemma*, the writer’s *attention to* their own and others’ *emotions*, and the *meaning-making* derived from the explored experience [55, 56]. Since we wanted to prepare students for their future patient interactions with our communication course, we added a sixth criterion to the rubric: *link to action*, according to Hung et al. [54] This criterion evaluates whether writers mentioned actions they would or would take to apply their learnings.

These six criteria were rated considering four levels of reflective capacity. The levels used in the REFLECT rubric were developed by Wald et al. [55], building on established frameworks for mapping reflection, such as the work of Mezirow [57] and Kember et al. [58]. The written reflections were scored on a scale ranging from 0 to 3 on four levels: habitual action or *nonreflective* = 0, thoughtful action or *introspection* = 1, *reflection* = 2, and *critical reflection* = 3 [16]. The sum of the six criteria of the rubric is the overall reflection score, the highest possible value of which is 18 (6 criteria × highest score 3) (cf. *Additional Information* for exemplified scorings). Two medical student assistants and the first author with a background in communication science were trained as raters, discussing the rubric’s criteria until reaching a shared understanding, as advised by previous research on the application of the REFLECT rubric [55, 56]. Subsequently, they analysed 50 written reflections collected in a pilot study (summer term 2020) under identical conditions until they reached a high level of interrater reliability (the intraclass coefficient [ICC] for a one-way random model with measures of absolute agreement was 0.95). The internal consistency of the ratings (6 items) was satisfactory, with Cronbach’s alpha of a positive effect of 0.74.

We received written reflections from 117 students from the three variants. Three students did not answer all nine reflection prompts; thus, their texts were excluded from the analysis. Each of the remaining 114 students wrote 9 reflections (3 prompts per module in 3 modules at different time points). All 1026 reflection prompts were analysed. Each module’s three reflection prompts were analysed jointly to ensure that the reflection process was pictured in its entirety while leaving the three core elements of the reflection (*awareness, understanding, and transfer*) recognisable. For each student, three overall scores were identified. For quality assurance, we repeated the interrater reliability measurement after 18.90% and 54.37% of the ratings, with the ICCs remaining high (0.94 and 0.87, respectively). SPSS (version 28) was used for statistical analysis. We used a mixed ANOVA to assess the effects of course variants on reflective capacity (as measured by reflection scores [RS]). RS were normally distributed for all groups, as assessed by the Shapiro-Wilk test (*p* >.05). We conducted post hoc analysis using Tukey’s corrected p-values to test for differences between the variants. Furthermore, we used the scoring scheme of the rubric to verify the levels of reflection and conducted a mixed ANOVA with a Creenhouse-Geisser correction to analyse whether the levels of reflection changed during the course in the three variants. Lastly, after analysing the scatterplots, a Kendall’s tau-b correlation coefficient was calculated to assess the linear relationship between RS and text lengths.

## Results

Our first research objective was to analyse which teaching approach is most effective in reflecting on the course content, comparing an e-learning course variant that focuses on independently deducing the course content (VR) with an explanatory course variant based on video modelling using good practice examples and exemplifications (VM) and a course variant merging both approaches (VMR).

### Comparison of the three e-learning variants

The comparison of the three e-learning variants showed a statistically significant interaction between time (module) and group (VM, VR, and VMR), Greenhouse–Geisser *F(3.649, 202.51) = 5.98*, *p* < .001, partial η² = 0.097, *as well as* a significant difference between the groups for Module 1, but none within the groups. Tukey post hoc analysis revealed a significant difference (*p* =.008) between RS of variants VM and VR in Module 1 (-1.90, 95% CI [-3.39, -0.41]). The mean RS in Module 3 was *M* = 14.22 (*SD* = 2.23) for VM, *M* = 13.56 (*SD* = 2.48) for VR, and *M* = 13.24 (*SD* = 2.21) for VMR. Regarding students’ RS at the end of the course, we could not determine significant differences between the variants at the end of the course in Module 3. Therefore, regarding our first research question about which variant was best in reflecting on the course content, we could not determine a variant that fostered reflection significantly better.

### Levels of reflection in each variant

Our second research objective was to determine which levels of reflection students achieved in the three e-learning variants. With means of RS between 11.44 and 14.22 (scale 0–18; Table 2), students reached the second highest level of four levels, the levels of *reflection*, in all variants and modules (m). Students in VM improved significantly during the course, with Greenhouse–Geisser of *F*(1.58, 60.00) = 23.96, *p* < .001, *partial η² = 0.387*, and Cohen’s *d* = 0.79, indicating a large effect [59]. This significant improvement occurred from Module 1 (*M* = 11.44 [*SD* = 3.11]) to Module 2 (*M* = 14.00 [*SD* = 1.99] *t*(38) = 6.57, *p* < .001, Cohen’s *d* = 1.05), with no significant differences between Modules 2 and 3 (*M* = 14.22 [*SD* = 2.23]). The ANOVA with a Greenhouse–Geisser correction showed no significant differences between the modules in VR (Module 1: *M* = 13.33 [*SD* = 3.02], Module 3: *M* = 13.56 [*SD* = 2.48]), *F*(1.65, 62.69) *= 0.13, p =*.836). Neither was there a significant difference in VMR during the course (Module 1: *M* = 12.56 [*SD* = 1.93); Module 3: *M* = 13.24 [*SD* = 2.21]), *F*(1.78, 62.55) = 1.40, *p* =.253). In summary, the only significant improvement during the course occurred in VM. Students of all course variants achieved the second-highest level of *reflection*.

**Table 2:** Reflection scores (RS) means achieved in the three course variants

	VM (*n* = 39)			VMR (*n* = 36)			VR (*n* = 39)		
	Module 1	Module 2	Module 3	Module 1	Module 2	Module 3	Module 1	Module 2	Module 3
Overall reflection score (mean)*, scale 0–18	11.44	14.00	14.22	12.56	13.17	13.24	13.33	13.46	13.56
SD	3.11	1.99	2.23	1.93	2.12	2.21	3.02	2.54	2.48
Minimum	6	10	9.5	9	10	9	5.5	6	7
Maximum	18	17.5	18	16	16	18	18	17.5	17

*Based on three reflection prompts in each module

### Correlation of text lengths with reflection scores

Our third research question explored whether length was related to quality. The most extended text was written in VM in Module 1 (578 words). Similarly, the variant VM of Module 1 showed the longest texts on average (*M* = 311 words, *SD* = 94.73). The shortest texts, on average, were composed in VR in Module 3 (*M* = 246 words, *SD* = 42.22). Variant VR also included the shortest text overall, written in Module 2 (160 words). The text length decreased from Module 1 to Module 3 (Table 3).

**Table 3:** Reflection scores (RS) and text lengths in words according to the three course variants

	VM (*n* = 39)			VMR (*n* = 36)			VR (*n* = 39)		
	Module 1	Module 2	Module 3	Module 1	Module 2	Module 3	Module 1	Module 2	Module 3
RS (mean)	11.44	14.00	14.22	12.56	13.17	13.24	13.33	13.46	13.56
Text length (mean)	311	266	253	282	251	256	264	261	246
SD	94.73	55.90	50.70	46.98	37.27	44.84	51.40	52.47	42.22
Text length (min)	198	191	193	172	186	196	177	160	192
Text length (max)	578	458	419	413	337	363	403	466	362

We also analysed the linear relationship between RS and text lengths (Table 4). The RS correlated significantly positively with each other across the modules. There were moderate effects between RS of Module 1 and RS of Module 2 (*r* =.369, *p* < .001) and small effects between RS of Module 2 and RS of Module 3 (*r* =.219, *p* =.001) and between RS of Module 1 and RS of Module 3 (*r* =.142, *p* =.039). Similarly, there were significant positive correlations within the text lengths across all modules, all with moderate effects: between Module 2 and Module 3 (*r* =.443, *p* < .001), between Module 1 and Module 2 (*r =*.414, *p* < .001), and between Module 1 and Module 3 (*r* =.304, *p* < .001). We also found significant positive correlations between RS and text lengths in all modules, with moderate effects between RS of Module 3 and text lengths of Module 3 (*r* =.404, *p* < .001) and RS of Module 2 and text length of Module 2 (*r* =.345, *p* < .001) and small effects between RS of Module 1 and text lengths of Module 1 (*r* =.235, *p* < .001).

**Table 4:** Kendall’s tau-b correlations between reflection scores (RS) and text lengths in the three modules, *N* = 114

		RS Module 1	RS Module 2	RS Module 3	Text length Module 1	Text length Module 2	Text length Module 3
RS Module 1	Kendall’s tau-b	1	0.369^**^	0.142^*^	0.235^**^		
	Sig. (2-tailed)		< 0.001	0.039	< 0.001		
RS Module 2	Kendall’s tau-b		1	0.219^**^		0.345^**^	
	Sig. (2-tailed)			0.002		< 0.001	
RS Module 3	Kendall’s tau-b			1			0.404^**^
	Sig. (2-tailed)						< 0.001
Text length Module 1	Kendall’s tau-b				1	0.414^**^	0.304^**^
	Sig. (2-tailed)					< 0.001	< 0.001
Text length Module 2	Kendall’s tau-b					1	0.443^**^
	Sig. (2-tailed)						< 0.001
Text length Module 3	Kendall’s tau-b						1
	Sig. (2-tailed)						

**Correlation is significant at the 0.01 level (2-tailed)

*Correlation is significant at the 0.05 level (2-tailed)

## Discussion and conclusions

Previous research has indicated that reflection can promote clinical communicative skills [5]. However, there are no clear best practices for embedding reflection into (online) medical education [31, 34, 35, 37]. Against this background, we analysed how reflection processes can be fostered in an e-learning course on medical communication competence. We examined how deeply students reflected on the course content, as well as on their personal learning process, by measuring students’ reflective capacity in three e-learning variants of the course with different proportions of reflection triggers.

With our first research question we analysed which e-learning course variant is most effective in reflecting on the course content. According to our data, at the end of the course, the quality of the students’ reflections did not differ significantly among the variants. In VM, students showed the lowest reflection score at the beginning but improved their reflective capacity significantly from Module 1 to Modules 2 and 3. By contrast, in VR and VMR, students started with higher RS and maintained a reflection level beyond description across all modules. Therefore, in VR and VMR, no significant developments were measured. This is consistent with previous research results showing that students exhibit limited and varied development of reflective skills through reflective writing [35].

In Module 1, there was a significant difference between the course variants. The design of the beginning of the course seems to have an effect. This significant difference between variant VM (without a reflection trigger) and VR (with two reflection triggers), considered in connection with the higher reflection values in Module 1 of VR compared to VM, indicates that the placement of reflection triggers at the beginning of the course creates the setting and awareness for reflection. Accordingly, a reason for the lack of improvement in VR and VMR could be that the course designs of these variants were more focused on reflection than the design of VM, and the students had to interpret some content themselves before they wrote their first reflection at the end of Module 1, which might have fostered their self-reflective attitude. Previous research on reflection has emphasized the importance of promoting a self-reflective attitude by encouraging students to reflect on and to evaluate their own learning [60]. In our variants VR and VMR, the open question at the beginning, in which students had to write about their previous knowledge or their personal thoughts on a topic in the field of medical communication, could have promoted a self-reflective attitude. This could have improved students’ reflective capacity before the first written reflection prompt.

Since our analysis examined how deeply the students reflected on the learning content and their learning process, their reflections could only be initiated at the end of a module. In VM, the first reflection prompt was written reflection at the end of Module (1) The RS of VM demonstrated that students particularly increased their level of reflection after this first written reflection from Module 1 to Module (2) Thus, the first reflection prompts might have already trained students’ reflective capacity, which would be consistent with the interpretation that the reflection-oriented learning environments of VR and VMR (with the open question at the beginning of the modules and, in VR, additionally with the interactive exercise as reflection triggers) could have already promoted students’ reflective capacity before the first measurement. This interpretation would indicate the effectiveness of the reflection triggers in VR and VMR, especially after their first use (Module 1), after which no more effects are recognisable. The latter would again be in line with previous research that considers the development of reflective skills through written reflection to be limited [35]. Further, the higher initial scores in VR and VMR might have limited the possible increase in RS.

Regarding our second research question and the overall level of reflection, the RS achieved in all nine measurements and variants showed that students consistently accomplished a medium level of reflection (the second-highest level of the rubric). This level goes beyond descriptive texts, indicating active engagement with the learning material and good reflection (cf. *Additional Information* for two examples of written reflections and their scoring). Lower levels of reflection are purely descriptive and reproductive, the medium level indicates personal insights beyond theory, and high level reflection involves critical reflection, including change of perspective and transformative learning [35, 55, 57, 61]. Our finding is contrary to pertinent literature stating that students mainly reflect on a descriptive level [15, 35, 61, 62] but is consistent with previous studies indicating that the highest level of reflection, *critical reflection*, is unlikely to occur frequently [57]. That students commonly do not reach the level of critical reflection was described in previous studies that analysed reflection exercises in health professions [63–65] as well as in other disciplines [61, 66–68].

There is no general agreement in the literature regarding which factors hinder students from developing high quality reflections and critical reflection. A possible cause could be that accomplishing critical reflection, the highest level of reflection, requires time [66, 69], as it involves perspective transformation, which might include a significant period from initial observations to final conclusions [57, 66]. To improve reflective capacity and to support critical reflection, recent literature has recommended teaching students the benefits of reflection. Students should understand that reflective practice can support them in their development [31] and should be provided with evidence of the potential educational and practice-related benefits of reflection [70]. High-quality reflection may also be fostered by outlining the components of critical reflection to students, such as linking past, present, and future experiences, integrating cognitive and emotional experiences, considering experiences from multiple perspectives, stating lessons learned, and planning future behaviour [70].

With our third research question, we aimed to clarify whether longer written reflections were more qualitative and should be encouraged, for example, through a specific number of characters or more guiding questions. A higher number of words could indicate that more aspects and arguments were mentioned, which is evaluated as a higher quality of reflection. However, long texts could be formulated repetitively without offering more content, and deep reflections could also be formulated concisely. Our findings show significant positive correlations between RS and text length, indicating that higher reflective quality could possibly be measured in longer texts. This is consistent with previous research results indicating that when students engage in higher levels of reflection, they tend to write longer reflections [71]. Prior studies have described that word count correlates with reflection scores [67]. Important questions about possible reasons for this remain unanswered in current literature. Ottenberg et al. have noted that longer reflections might be assumed to show profound reflection, but that this may not always be the case. They therefore suggest that the contents of reflections could be examined as reliable evidence of depth of reflection [67].

### Limitations and future research

Although our study provides new insights and evidence, we acknowledge some limitations. Based on these results, we could not derive whether the good reflective capacity levels demonstrated by the students’ written reflections led to better communicative competence and skills in patient care. We also noticed three factors that might have limited the increase in RS. First, a feedback questionnaire provided to all students at the end of the course showed that students perceived the module themes *communicative basics* (Module 1) and *structure of the conversation* (Module 2) as less challenging than *emotion and empathy* (Module 3). Increasing difficulty may have distorted the findings. RS, which remained constant as difficulty increased, might have been higher with steady difficulty.

Second, habituation effects must be considered: The reflection prompts were provided with the same wording across all course modules to ensure comparability and because previous research has proven single samples to be less suitable for accurate reflectivity assessments [51]. Nevertheless, students might have been less motivated to compose qualitative answers towards the end of the course, as they had to answer the same questions three times. To analyse this potential effect, time logs could verify whether students spent less time on the reflection texts over the course and whether this influenced RS.

Third, teaching students the basics and importance of reflection before the reflection exercises seems to improve their reflection [31, 70]. In most studies, participants were trained in advance [72]. We did not pre-train students in this study, nor did we teach them the importance of reflection, as this might have distorted the results of our research questions. We also did not provide feedback to our students, as it could have influenced their motivation and the results of the study. However, there is broad evidence that ongoing feedback can improve reflection [70, 73, 74], and we plan to incorporate it into our subsequent courses. Since feedback given by lecturers requires resources, additional future possibilities could be the complementary use of automated feedback and more advanced artificial intelligence technologies to analyse and assess reflective writing, as well as to provide personalised feedback to students [68].

Lastly, future research will have to clarify the development of reflective skills over time and to prove whether and how increased reflective capacity leads to better physician–patient interactions in medical practice. There is limited research on the relationship between the quality of reflections and academic achievements of medical students [67], and previous studies have shown mixed results [75] or reported little evidence on how reflection correlates with other measures or performances in medical school [67, 76].

### Practice implications

Our study contributes to the understanding of how reflection can be embedded and promoted in online learning environments, offering a teaching strategy leading to levels of reflection beyond the descriptive level with the aim of improving physician–patient interactions. The developed reflection prompts can be embedded easily into e-learning and enable qualitative reflections even in short time frames if little teaching time is available. The teaching approach of starting the course with an open-ended question promoting reflection led to good reflection levels right from the beginning, presumably because these questions sharpened the awareness of reflection.

Since students achieved a good level of reflective capacity in all three course variants, and no variant promoted reflection significantly better, we recommend choosing the variant adapted to the needs of the course. If there is a demand for a more interactive learning environment from faculty or students, VR can be used as a suitable variant. If students need more exemplifications, VM can be used, as video modelling has proven to be effective for the training of communicative competence in previous research results [4, 40].

### Electronic supplementary material

Below is the link to the electronic supplementary material.

Supplementary Material 1

## Data Availability

The datasets used and/or analysed during the current study are available from the corresponding author upon reasonable request.

## Abbreviations

ICC: intraclass correlation coefficient
M: mean
m1/m2/m3: module 1/module2/module3
RS: reflection score
SD: standard deviation
SEM: standard error mean
VM: video modelling
VR: video reflection
VMR: video modelling and reflection
