# Supplementary material for: Reflect to interact – fostering medical students’ communication through reflection-focused e-learning
Source: BMC Med Educ. 2024 May 15;24:541. doi: 10.1186/s12909-024-05368-4 (PMC11097513; doi:10.1186/s12909-024-05368-4)
Supplement: Supplementary file 1 — Supplementary Material 1 [file 12909_2024_5368_MOESM1_ESM.pdf]

Supplementary Material

Examples of two written reflections and their scoring

Text A (VM, m1); see original German version below

The basis for a good doctor-patient relationship or for the following conversation/anamnesis is already laid during the greeting. If a good greeting is successful, the subsequent working atmosphere is much more pleasant as there are no longer any open questions regarding the form and basic conditions of the conversation. By means of the negative examples shown, it becomes obvious how the patient can be unsettled by such "misbehaviour". Timewise, you can hardly notice any differences.

Humans communicate in many ways. If a person, especially in the role of the doctor, is not aware of this fact, problems can arise in a conversation that you cannot resolve or overcome. However, if you are aware of this, it can be better reflected on whereby a conversation failed or how it can be optimized. A patient usually comes with a concern and may be insecure, depending on the situation. If you do not manage to create an appropriate atmosphere at the beginning of the conversation, it can become difficult to hear about, for example, intimate matters which could significantly contribute to the diagnosis.

It is important to pay attention to language, gestures, facial expressions, and tone of voice in a conversation, as conversations can thereby essentially be influenced. Due to the immense importance of anamnesis for the diagnosis, it is worthwhile to increasingly delve into this topic. For future conversations, I would primarily plan to prepare and optimize different forms of beginnings of conversations. It is also very important to reflect on conversations that went less ideal in order to find out why possible disruptions occurred.

Scoring of text A

The following written reflection, text A, from variant VM, m1, addresses several aspects which influence doctor-patient communication: the greeting, the atmosphere (which can be influenced by the greeting), and different types of communication. Both raters therefore awarded 2 points (0-3) for the criterion *writing spectrum*. The discussion of the aspects mentioned remains largely superficial: For instance, the student writes that it can lead to difficulties in conversation "if you do not manage to create an appropriate atmosphere at the beginning of the conversation" but does not mention how an appropriate atmosphere could be created. It is also stated that "humans communicate in many ways" and that "if a person, especially in the role of the doctor, is not aware of this fact, problems can arise (...)", but without going into detail at this point, in which ways communication can take place and what the doctor should be aware of. Since the *description of conflict/issue* is not extensive, it was rated 1 point by both raters.
The *meaning-making* contains approaches to reflection, but overall, it does not go into depth. For instance, the student concludes that "if you do not manage to create an appropriate atmosphere at the beginning of the conversation, it can become difficult to hear about, for example, intimate matters which could significantly contribute to the diagnosis." The writer also considers that "it is important to pay attention to language, gestures, facial expressions, and tone of voice as conversations can thereby essentially be influenced." Nevertheless, relatively few conclusions are drawn, and there is no in-depth analysis, so both raters awarded 1 point for *meaning making*. Emotions on the part of the doctor or the patient are not discussed, so both raters awarded 0 points for *attending to emotions*. In the last two sentences, references to the writer's plans for

future actions are visible ("I would primarily plan to..."). Though, in the last sentence, these are formulated in general and in the passive voice ("It is also very important to reflect (...)") and not explicitly or in the first-person perspective. It remains vague as to what is specifically important for the reflection and what "less ideal" and "possible disruptions" specifically mean. Rater 1 awarded 2 points for *link to action*, rater 2 awarded 1 point. Overall, the individual perspective of the author is partially and to a limited extent recognizable in the text, so that *writer's presence* was rated with 1 point by both raters.
With an overall score of 6.5 (rater 1: 7 points, rater 2: 6 points) out of 18 possible points, the text remains on a lower descriptive level.

**Criteria (REFLECT rubric)**	**Rater 1**	**Rater 2**	**Ø**
Writing spectrum	2	2	2
Meaning making	1	1	1
Link to action	2	1	1,5
Description of conflict/issue	1	1	1
Attending to emotions	0	0	0
Writers' presence	1	1	1
**Total reflection score**	**7**	**6**	**6,5**

Text B (VR, m3); see original German version below

I have tried to put myself in the patient's position and then consider the doctor's corresponding reactions as if they were for me (as a patient). In doing so, I noticed that even small changes in communication can cause significant differences in the effect. The choice of words, which you often make mainly intuitively in conversations, plays a very important role in reacting to emotions.

Among other things, I have learned how empathy is (theoretically) defined and that in its expression a cognitive, an affective, and a behavioural component always play a role. Even if this is theoretical at first, I hope, thereby, also to be able to implement the practical "application" of empathy more consciously and better in the future. This is especially useful because, on the one hand, it strengthens/improves the relationship between doctor and patient and thereby also the compliance is increased. Additionally, patients are more open in their communication and can better approach you with their problems if you have shown in previous conversations that you can react empathetically to emotions that may have been unpleasant.

According to the description in the previous reflection section, I will intend to keep in mind the relevance of situationally appropriate reactions to emotions on the part of patients for my future occupation as a doctor, and I will further practice both non-verbally, especially through facial expressions, and with words, to name the emotions and moods present in the room and to demonstrate my understanding as well as to offer my support. Furthermore, and I think I still have much to learn in this regard, I resolve to also become aware of my own emotions, with which I start a conversation or which arise during a conversation, in order to not negatively "fall victim to" the principle of countertransference, for instance. Not to be forgotten are the causes of non-compliance. I hope that in the future and in upcoming conversations and treatments of patients, I

do not forget that non-compliance can have varied causes, which can also be due to me as a doctor.

Scoring of Text B

The following text B from variant VR, m3, is an example of a written reflection that goes beyond the descriptive level. It was rated 17 out of 18 points by both raters. The only category which did not receive the highest score from both raters is *writing spectrum* since the reflection could have included more topics. The *description of conflict/issue* is relatively extensive, for example, at the point: "I noticed that even small changes in communication can cause significant differences in the effect. The choice of words, which you often make mainly intuitively in conversations, plays a very important role in reacting to emotions." With regard to *meaning making*, several profound conclusions are drawn, for instance, when considering empathy: "it (the "application of empathy") strengthens/improves the relationship between doctor and patient and thereby also the compliance is increased. Additionally, patients are more open in their communication and can better approach you with their problems (...)". Additionally, the author attempts a change of perspective ("I have tried to put myself in the patient's position and then consider the doctor's corresponding reactions as if they were for me as a patient".) Regarding *attending to emotions*, it is noticeable that no specific emotions are named in this reflection. However, the student discusses how important the choice of words is when reacting to (patient's) emotions. Also, own emotions are discussed in more detail: "I resolve to also become aware of my own emotions, with which I start a conversation, or which arise during a conversation, in order to not negatively "fall victim to" the principle of countertransference, for instance". The student's own emotions are also part of the discussed future actions/*links to action*, which are specifically described: "I will further practice, both non-verbally, especially through facial expressions, and with words, to name the emotions and moods present in the room and to demonstrate my understanding as well as to offer my support.” In addition, explicit self-reflection can be seen in this last paragraph: "I think I still have much to learn in this regard (...)" and the *writer's presence* clearly recognizable: "I hope (...) I do not forget that non-compliance can have varied causes, which may also be due to me as a doctor."

**Criteria (REFLECT rubric)**	**Rater 1**	**Rater 2**
Writing spectrum	2	2
Meaning making	3	3
Link to action	3	3
Description of conflict/issue	3	3
Attending to emotions	3	3
Writers' presence	3	3
**Total reflection score**	**17**	**17**

Text A, original German version

Die Grundlage für ein gutes Arzt-Patienten-Verhältnis beziehungsweise für das folgende Gespräch/die Anamnese wird schon bei der Begrüßung gelegt. Gelingt eine gute Begrüßung so ist die folgende Arbeitsatmosphäre deutlich angenehmer, da keine offenen Fragen mehr im Raum stehen, die die Form und Rahmenbedingungen des Gesprächs angehen. An den gezeigten Negativbeispielen wird deutlich wie der Patient durch solches "Fehlverhalten" verunsichert werden kann. Zeitlich gesehen kann man auch kaum Unterschiede erkennen.

Der Mensch kommuniziert auf viele Art und Weisen. Ist einer Person, vor allem in der Rolle des Arztes, diese Tatsache nicht bewusst, so kann es in einem Gespräch zu Problemen kommen, die man nicht bewältigen oder aufarbeiten kann. Ist man sich dessen jedoch bewusst, so kann besser reflektiert werden, woran ein Gespräch gescheitert ist bzw. wie dieses optimiert werden kann. Ein Patient kommt in der Regel mit einem Anliegen und kann je nachdem verunsichert sein. Schafft man es beim Gesprächseinstieg nicht eine angemessene Atmosphäre herzustellen, kann es schwierig werden z.B. intime Dinge zu erfahren, die wesentlich zur Diagnosestellung beitragen könnten.

Es ist wichtig auf Sprache, Gestik, Mimik und Tonlage zu achten in einem Gespräch, da Gespräche wesentlich dadurch beeinflusst werden können. Wegen der enormen Bedeutung der Anamnese für die Diagnosestellung lohnt es sich mit diesem Thema vermehrt auseinanderzusetzen. Für zukünftige Gespräche würde ich mir vor allem vornehmen verschiedene Formen des Gesprächseinstiegs vorbereiten und zu optimieren. Sehr bedeutend ist es auch über weniger optimal gelaufene Gespräche zu reflektieren, um herauszufinden, weshalb etwaige Störungen aufgetreten sind.

Text B, original German version

Ich habe versucht, mich an der Stelle des Patienten zu sehen und dann die entsprechenden Reaktionen der Ärztin so zu nehmen, als wären sie für mich (als Patient). Dabei ist mir aufgefallen, dass bereits kleine Änderungen in der Kommunikation erhebliche Unterschiede in der Wirkung hervorrufen können. So spielt die Wortwahl, die man in Gesprächen ja häufig vor allem intuitiv trifft, bei der Reaktion auf Emotionen eine sehr wichtige Rolle.

Unteranderem habe ich gelernt, wie Empathie (theoretisch) definiert wird, und dass in ihrem Ausdruck immer eine kognitive, eine affektive und eine behaviorale Komponente eine Rolle spielt. Auch wenn dies erst einmal theoretisch ist, hoffe ich, dadurch auch die praktische "Anwendung" von Empathie in Zukunft bewusster und besser umsetzen zu können. Das ist vor allem deshalb nützlich, weil so zum einen die Beziehung zwischen Arzt und Patient gefestigt/gebessert wird und damit auch die Compliance erhöht wird. Außerdem sind Patienten offener in Ihrer Kommunikation und können sich mit ihren Problemen besser an einen wenden, wenn man in vergangenen Gesprächen gezeigt hat, dass man empathisch auf Emotionen, die eventuell auch unangenehm waren, reagieren konnte.

Gemäß der Beschreibung im vorherigen Reflexionsabschnitt werde ich mir für meine spätere Tätigkeit als Arzt vornehmen, die Relevanz von situativ angemessenen Reaktionen auf Emotionen seitens der Patienten im Sinn zu behalten und weitergehend üben, sowohl nonverbal, vor allem über Mimik, wie auch mit Worten, die im Raum stehenden Emotionen und Stimmungen zu benennen und sowohl mein Verständnis zu zeigen, wie auch meine Unterstützung anzubieten. Außerdem, und ich denke in diesem Punkt muss ich noch einiges lernen, nehme ich mir vor, mir

auch meiner eigenen Emotionen bewusst zu werden, mit denen ich in ein Gespräch gehe oder die im Laufe eines Gespräches aufkommen, um beispielsweise nicht dem Prinzip der Gegenübertragung negativ "zum Opfer zu fallen". Nicht zu vergessen sind die Ursachen für Non-Compliance. Ich hoffe, dass ich in Zukunft und in den kommenden Gesprächen und Behandlungen von Patienten nicht vergessen, dass Non-Compliance vielfältige Ursachen haben kann, die auch an mir als Arzt liegen können.
